# The moderating role of legal emotions in the relationship between sensation seeking and risk-taking behaviors among college students

**DOI:** 10.3389/fpsyg.2025.1605528

**Published:** 2025-07-24

**Authors:** Zhiqiang Wang, Min Zhang, Shuhui Xu

**Affiliations:** ^1^School of Teacher Education, Taizhou University, Zhejiang, China; ^2^Institute of Higher Education, Wenzhou University, Zhejiang, China

**Keywords:** sensation seeking, legal emotions, risk-taking behaviors, legal education, college students

## Abstract

The dual-system model proposes that asynchronous maturation of the socioemotional and cognitive control systems underlies the high incidence of adolescent risk-taking. Although sensation seeking is strongly linked to such behaviors, the role of social emotions-particularly legal emotion-remains underexplored. In this study, 127 university students completed a sensation-seeking scale and the College Students’ Legal Emotions Questionnaire. A subset of 110 participants with valid survey data then undertook an objective behavioral assessment of risk-taking (Balloon Analogue Risk Task). Results indicated that sensation seeking significantly and positively predicted risk-taking propensity, and that positive legal emotions attenuated this effect, whereas negative legal emotions showed no moderating impact. These findings highlight a novel intervention target: fostering positive legal emotions via school-based legal education may effectively reduce adolescent risk-taking behaviors.

## Introduction

Risk-taking behavior is a decision-making process where individuals, despite being aware of potential risks, engage in actions driven by the desire for favorable outcomes (Ben-Zur and Zeidner, 2009). Early research viewed adolescent risk-taking as maladaptive behavior, leading to the development of problem behavior theory at the behavioral level (Jessor and Jessor, 1977). Studies have shown that adolescents are more prone to engage in risk-taking behaviors such as smoking, reckless driving, and excessive drinking, all of which can negatively impact their healthy development (Chein et al., 2011). Sensation seeking, defined as the pursuit of novel, varied, complex, and intense experiences through risk-taking behaviors across different domains (Mann et al., 2017), has been shown to predict problem behaviors in adolescents. Research also highlights the influence of sensation seeking on deviant peer associations and bullying behavior (Cui et al., 2016). In other words, sensation-seeking tendencies can drive adolescents to engage in various risk-taking behaviors (Liu et al., 2025). Legal emotions, as a subcategory of emotions, refer to an individual’s complex response to legal stimuli, such as the spirit of the rule of law, the current legal system, and its operation—play a critical role in shaping adolescents’ normative judgments and physiological arousal when confronted with risky situations. By influencing both moral evaluations and autonomic nervous system activation, legal emotions can either deter or exacerbate adolescents’ propensity to take risks, making them a central focus of the present study (Xu et al., 2024; Jianhua et al., 2025). Thus, understanding the mechanisms behind adolescent risk-taking behaviors is crucial for promoting their healthy development.

### Sensation seeking and risk-taking behavior

The dual-system model is commonly used to explain risk-taking behavior, suggesting that the adolescent brain’s reward-seeking, emotion-related system and the impulse-control system mature at different rates. This disparity in maturation contributes to the high incidence of risk-taking behaviors during adolescence (Steinberg, 2017). Based on the Expected-Value-of-Control (EVC) theory (Shenhav et al., 2013), some researchers have conceptualized risk-taking behavior and control attributes as orthogonal dimensions: the horizontal axis represents risk-taking, ranging from non-risky to risky behavior, while the vertical axis represents control, from habitual to effortful behavior (Do et al., 2020). In other words, adolescents’ engagement in risk-taking behaviors depends on cognitive control. Further research indicates that the dual-pathway model is dynamic, with cognitive development potentially shifting adolescents from maladaptive to adaptive risk-taking (Yu and Hu, 2023).

Sensation seeking is a personality trait characterized by a desire for novel and complex sensations. Sensation seekers are willing to take physical and social risks for stimulation and expect non-monetary rewards to validate their actions (Zuckerman, 1983). The heightened hormone secretion in adolescents increases the brain’s sensitivity to rewards, driving them to engage in risky behaviors to satisfy their need for novel and stimulating experiences (Shulman et al., 2016). High sensation seekers show greater arousal, attention bias, and cognitive processing advantages in detecting risky signals, which may explain their tendency toward risk-taking (Tang et al., 2022). Research also found that deviant peers’ influence on high-risk aquatic behaviors increases with sensation-seeking levels (Shi et al., 2019). High sensation seekers are more likely to engage in dangerous activities, while low sensation seekers tend to avoid them. Thus, high sensation seeking leads to increased risk-taking behaviors and negative outcomes (Cheng et al., 2012).

Based on this, the study proposed the hypothesis.

*H1*: Sensation seeking can significantly and positively predict the tendency for risk-taking behavior.

### The moderating role of legal emotions

Previous studies have explored risk-taking behavior from various perspectives, but few have examined it through the lens of legal emotions. Rudolf von Jhering, a pioneer in the sociology of law, introduced the concept of “legal sentiment” (or “legal emotion”), distinguishing between objective legal sentiment as the source of law and subjective legal sentiment as closely tied to individual personality (Yan, 2012). Legal emotion, a subset of emotion, refers to an individual’s complex response to legal stimuli, including the spirit of the rule of law, the legal system, and its operations. This response reflects psychological phenomena linked to personal desires and needs. Legal emotion can be categorized as positive or negative. Positive legal emotion arises when the spirit of the law or the legal system aligns with an individual’s expectations, leading to a pleasant subjective experience. It reflects recognition and identification with the law, and is encouraged through societal education. Negative legal emotion, on the other hand, involves feelings such as disappointment, contempt, or aversion toward the law or its operation (Xu et al., 2024).

The purely rational perspective has proven unrealistic in real life, as emotions play a significant role in many behaviors, including criminal behavior, a form of antisocial conduct (Blair, 2017; Dippong and Fitch, 2017). Some scholars argue that positive emotions encourage risk-taking, while negative emotions promote risk aversion. According to the Affective Generalization Hypothesis, positive emotions make individuals perceive risks as lower, leading to increased risk-seeking behavior. Conversely, negative emotions generally lead to risk avoidance. However, the Mood Maintenance Model suggests that individuals in negative emotional states may seek risks to generate positive emotions and alleviate their negative mood (Devlin et al., 2015). Research has shown that specific emotions influence risk-taking: anger increases risk-seeking, anxiety promotes risk aversion, and sadness encourages high-risk, high-reward decisions (Ferrer et al., 2017; Aslan et al., 2017). Previous studies primarily examined the influence of emotions on risk-taking from the perspective of emotional valence (Yu and Hu, 2023). However, little research has focused on the effects of positive and negative legal emotions on risk-taking behavior. Studies have found that awe influences decision-making, enhances happiness, and promotes prosocial behavior (Rui et al., 2013). Additionally, awe has been shown to reduce moral risk-taking behavior (Ming et al., 2019). One reason individuals comply with the law is the sense of awe it evokes. Awe, a positive legal emotion, likely inhibits risk-taking behavior.

Emotions directly influenced risk-seeking behavior. According to the mood repair hypothesis, when individuals were in a negative emotional state, they sought risks in hopes of obtaining high rewards to improve their current negative emotional state (Li et al., 2016). As a subordinate concept of emotion, does negative legal emotion enhance the relationship between sensation seeking and risk-taking behavior? Based on this, the study proposed the following hypotheses:

*H2*: Positive legal emotion weakens the relationship between sensation seeking and risk-taking behavior.

*H3*: Negative legal emotion enhances the relationship between sensation seeking and risk-taking behavior.

## Methods

### Participants

This study initially recruited 127 undergraduates from a university in southern China. All participants first completed the Sensation Seeking Scale and the College Students’ Legal Emotions Questionnaire; after excluding 17 invalid surveys (e.g., incomplete or failed attention checks), 110 students remained. These 110 then performed the Balloon Analogue Risk Task (BART); after removing 6 datasets for task interruptions or outlier performance, the final sample comprised 104 participants (51 males, 53 females). Of these, 42 were sophomores and 62 juniors; 66 came from rural areas and 38 from urban areas. Fathers’ education levels were: 20 primary school or below, 38 middle school, 27 high school/vocational, 17 college, and 2 postgraduate. Mothers’ education levels were: 22 primary school or below, 42 middle school, 21 high school/vocational, 17 college, and 2 postgraduate. All participants had normal (or corrected) vision and no history of neurological or psychiatric disorders. Informed consent was obtained from each participant.

### Research procedure

The survey was conducted in groups, with graduate students in psychology acting as test administrators. During class time, participants completed demographic information, the Sensation Seeking Scale, and the College Students’ Legal Emotions Questionnaire via Tencent Questionnaire, taking approximately 8–12 min.

Risk-taking behavior was assessed using a behavioral task rather than as an experimental manipulation. Due to the large sample, the Balloon Analogue Risk Task was administered in cohorts of 15–20 participants. Prior to the task, participants received standardized instructions and completed three practice trials to familiarize themselves with the procedure. The task was programmed and presented using E-Prime 2.0 software. Upon finishing the formal task, participants received a small reward and were thanked for their participation. No variables were manipulated, and no control or treatment groups were formed; thus, the study follows a non-experimental, correlational design employing both self-report scales and behavioral indicators.

### Measure

#### Chinese version of the Brief Sensation Seeking Scale

The Chinese version of the Brief Sensation Seeking Scale (Chen et al., 2013) includes four dimensions—stimulation and adventure seeking, experience seeking, disinhibition, and boredom susceptibility—across 8 items, rated on a 5-point Likert scale (1 = strongly disagree to 5 = strongly agree). Higher scores reflect greater sensation seeking. In this study, Cronbach’s *α* = 0.67.

#### College Students’ Legal Emotions Questionnaire

Developed by Xu and Yan (2022), the College Students’ Legal Emotions Questionnaire comprises 33 items divided into two distinct subscales: an 11-item Positive Legal Emotions subscale (e.g., “I believe that under the protection of law, society will become increasingly better.”) and a 22-item Negative Legal Emotions subscale (e.g., “The credibility of law in social life is not very good.”). All items are rated on a 5-point Likert scale (1 = strongly disagree to 5 = strongly agree), with higher scores indicating stronger positive or negative legal emotional responses, respectively. In the present study, internal consistency was high, with Cronbach’s *α* = 0.90 for the Positive Legal Emotions subscale and α = 0.92 for the Negative Legal Emotions subscale.

#### Balloon Analogue Risk Task

**Figure 1:**
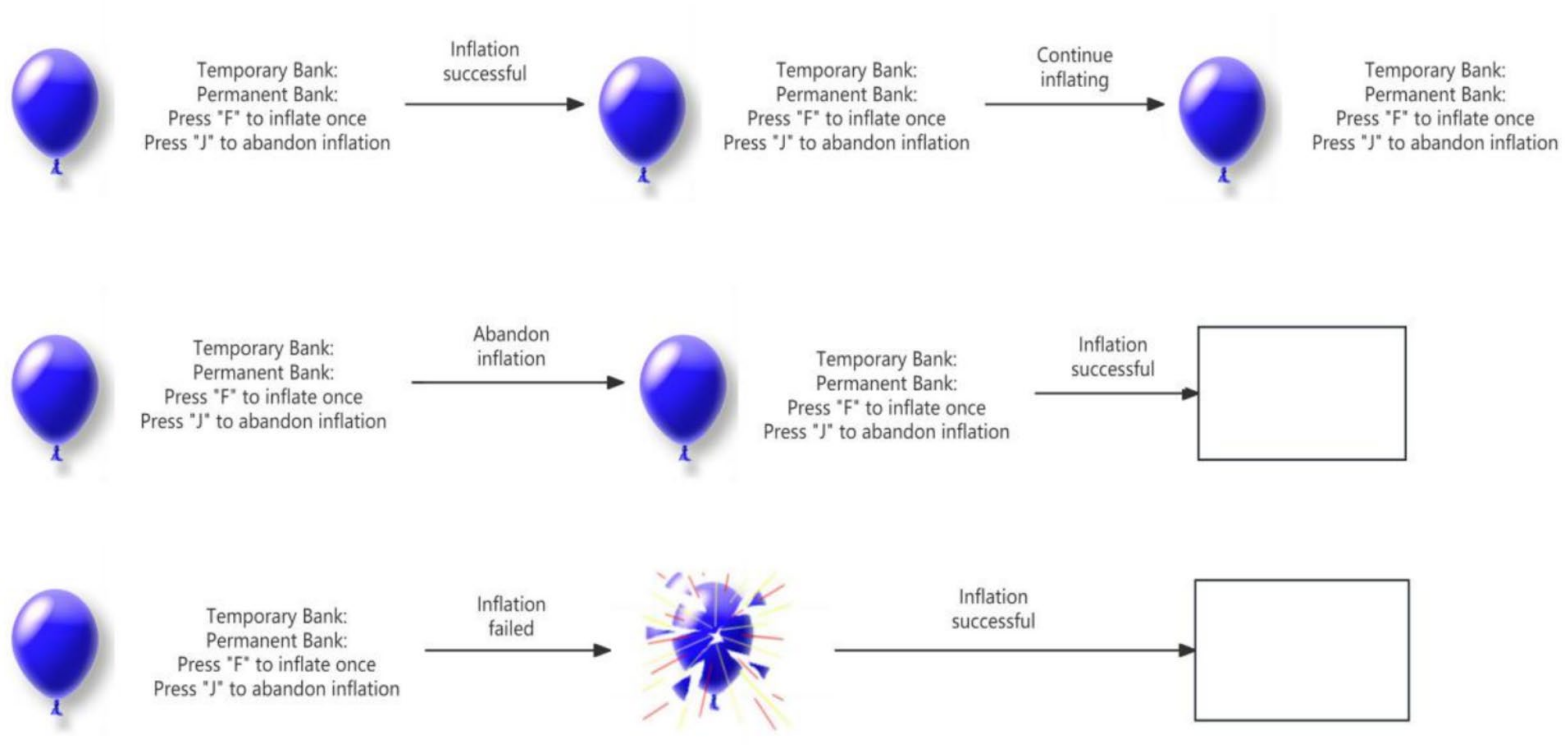
Schematic of the Balloon Analogue Risk Task.

To obtain an objective behavioral indicator of risk taking, we used the Chinese-adapted Balloon Analogue Risk Task (Lejuez et al., 2002). Implemented in E-Prime 2.0, the task began with three practice balloons—during which the experimenter explicitly informed participants that each balloon could explode after any number of inflations between 1 and 128—followed by 20 test balloons. On each trial, a deflated balloon appeared on screen alongside two counters: a temporary bank (current trial earnings) and a permanent bank (cumulative earnings). Participants pressed “F” to inflate (earning ¥0.01 real yuan per pump, with all earnings tallied and paid via WeChat transfer after the session) or “J” to cash out (transferring the temporary bank to the permanent bank and proceeding to the next balloon). The explosion threshold for each balloon was randomly drawn from a uniform distribution between 1 and 128 inflations, ensuring equal probability for each possible pump count. No between-group comparisons were made; the task served exclusively as an assessment tool. Risk-taking propensity was indexed by the average number of pumps on unexploded balloons (total pumps/number of intact balloons), with higher values indicating greater risk taking (see Figure 1).

### Analytic strategy

In this study, SPSS 27.0 was used to manage, store, and analyze the collected data. The statistical methods primarily included descriptive statistics, correlation analysis, and moderation effect testing, with the moderation effect tested using Process 4.2. Harman’s single-factor test was applied to examine common method bias in the questionnaire data. The results showed that the first common factor explained 27.71% of the variance, which is less than the critical threshold of 40%, indicating that there is no serious common method bias in the data of this study.

## Results

### Descriptive statistics and correlation analysis of sensation seeking, legal emotions, and risk-taking behavior

Descriptive statistics and correlation analysis were conducted for sensation seeking, positive legal emotions, negative legal emotions, and college students’ risk-taking behavior, with the results shown in Table 1. As can be seen from the table, sensation seeking was significantly positively correlated with college students’ risk-taking behavior (*r* = 0.49, *p* < 0.001). There were no significant correlations between positive legal emotions, negative legal emotions, and risk-taking behavior (*p* > 0.05), nor between these variables and sensation seeking (*p* > 0.05).

**Table 1:** Descriptive statistics and correlation analysis of sensation seeking, legal emotions, and risk-taking behavior (*N* = 104).

Variable	M	SD	1	2	3	4
1. Sensation seeking	3.02	0.51	1.00			
2. Positive legal emotion	4.20	0.49	0.07 (ns)	1.00		
3. Negative legal emotion	1.76	0.49	0.06 (ns)	−0.47^***^	1.00	
4. Risk-taking behavior	58.96	40.19	0.49^***^	−0.19 (ns)	0.13 (ns)	1.00

^***^p < 0.001, ns, non-significant (*p* > 0.05).

### Moderating effect of legal emotions

The moderating effect was tested using the Process macro (Model 1) for moderation analysis, with all variables standardized prior to analysis. The results are presented in Table 2. After controlling for parental education level, grade, and gender, sensation seeking was found to significantly positively predict risk-taking behavior. The interaction term between sensation seeking and positive legal emotions significantly negatively predicted risk-taking behavior (*β* = −0.075, *t* = −4.741, *p* < 0.001), indicating that positive legal emotions moderated the relationship between sensation seeking and risk-taking behavior. On the other hand, the interaction term between sensation seeking and negative legal emotions did not significantly predict risk-taking behavior (*β* = 0.011, *t* = 0.113, *p* > 0.05), suggesting that negative legal emotions did not moderate the relationship between sensation seeking and risk-taking behavior.

**Table 2:** Moderating effect analysis of legal emotions.

Dependent variable	Model 1		Model 2	
	Risk-taking behavior		Risk-taking behavior	
	*β*	*t*	*β*	*t*
Constant	0.534	0.782	0.688	0.886
Gender	−0.082	−0.376	0.008	0.031
Grade	−0.065	−0.286	−0.114	−0.444
Father’s education level	−0.130	−1.186	−0.090	−0.725
Mother’s education level	0.008	0.080	−0.075	−0.626
Sensation seeking	0.584	6.853^***^	0.532	5.298^***^
Positive legal emotion	−0.019	−1.222		
Negative legal emotion			0.097	1.077
Sensation seeking × positive legal emotion	−0.075	−4.741^***^		
Sensation seeking × negative legal emotion			0.011	0.113
*R* ^2^	0.432		0.273	
*F*	10.427^***^		5.152^***^	

^***^p < 0.001, ns, non-significant (*p* > 0.05).

**Figure 2:**
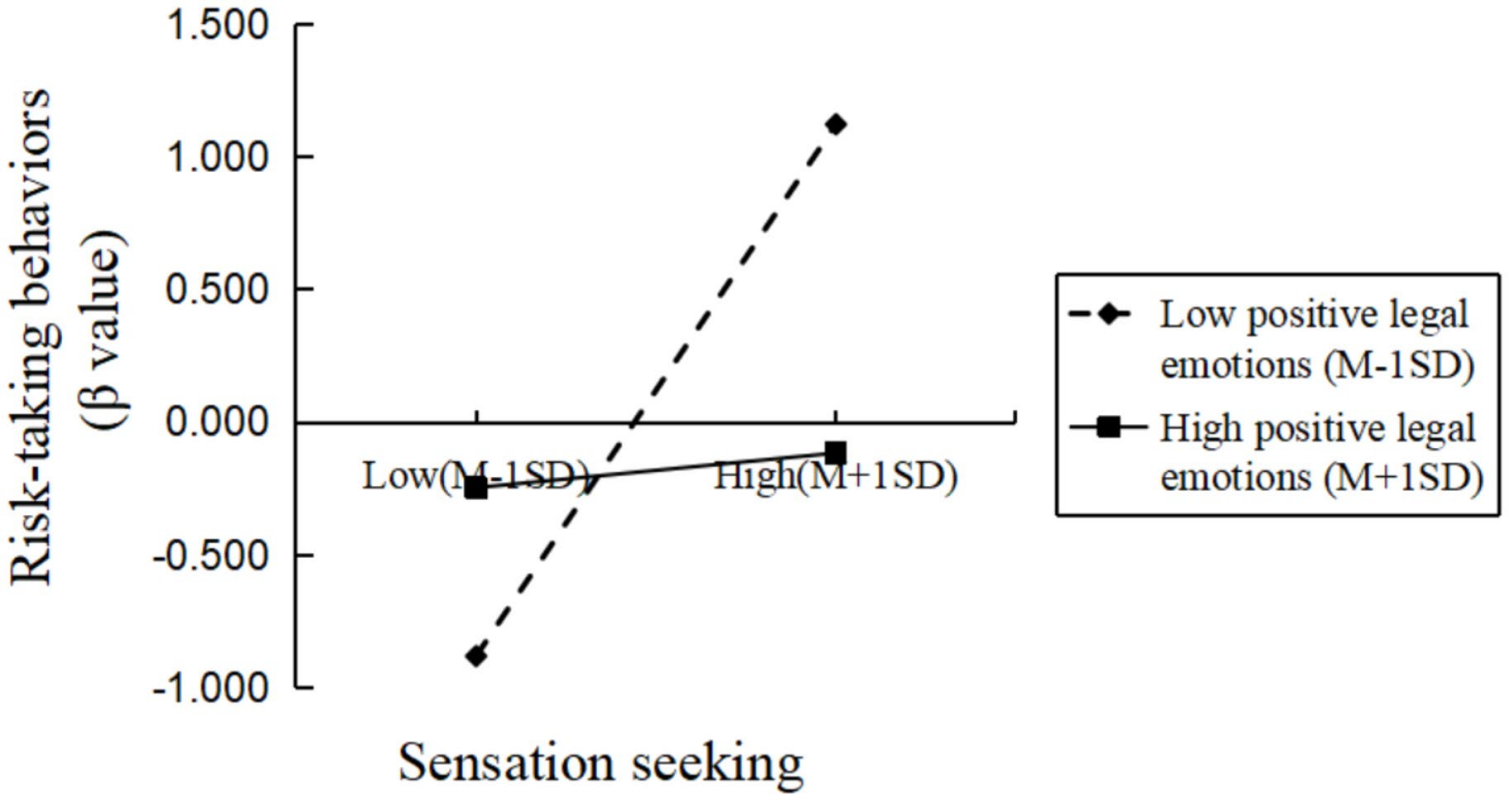
Moderating effect of positive legal emotions on the relationship between sensation seeking and risk-taking behavior in college students.

For positive legal emotions, which showed a moderating effect, further simple slope analysis revealed that at low levels of positive legal emotions (M − 1 SD), sensation seeking significantly positively predicted college students’ risk-taking behavior (*β*_simple = 0.989, *p* < 0.001). However, at high levels of positive legal emotions (M + 1 SD), sensation seeking did not significantly predict college students’ risk-taking behavior (*β*_simple = 0.177, *p* > 0.05). These results suggest that the lower the level of positive legal emotions, the stronger the predictive effect of sensation seeking on risk-taking behavior, and that positive legal emotions effectively alleviate the impact of sensation seeking on risk-taking behavior (see Figure 2).

## Discussion

This study found a significant positive correlation between sensation seeking and college students’ risk-taking behavior, indicating that sensation seeking was a direct factor influencing risk-taking behavior. Sensation seeking promoted risk-taking behavior among college students, which was consistent with previous studies (Lydon-Staley et al., 2020; Lydon-Staley and Geier, 2017). According to the dual systems model, high sensation seekers exhibit heightened socioemotional reward sensitivity alongside relatively immature prefrontal inhibitory control, reducing their capacity to regulate impulses and thereby increasing engagement in risky behaviors (Duell et al., 2016). Hypothesis 1 was thus supported.

This study found that positive legal emotions moderated the relationship between sensation seeking and risk-taking behavior. Specifically, as positive legal emotions increased, the predictive effect of sensation seeking on risk-taking behavior decreased and eventually became non-significant, effectively alleviating the impact of sensation seeking. Thus, Hypothesis 2 was supported.

According to Self-Determination Theory, intrinsic motivation and autonomous decision-making arise when needs for autonomy, competence, and relatedness are satisfied (Deci, 1971). Positive legal emotions—reflecting strong recognition of legal norms—first provide a sense of security and predictability, satisfying individuals’ perceived behavioral control; next, this security enhances feelings of competence in one’s decision-making; finally, elevated competence bolsters autonomy, enabling individuals to regulate impulses and choose non-risky actions even under high sensation-seeking drives.

Life History Theory suggests that individuals in resource-abundant environments adopt a slow life strategy characterized by long-term planning and risk aversion, whereas those in resource-scarce contexts adopt a fast life strategy marked by impulsivity and risk taking (Simpson et al., 2012). Fast strategy individuals are more prone to norm violations and impulsive behaviors (Liang and Yang, 2024). The maturation of a rule-of-law society symbolizes collective resource abundance, thus evoking positive legal emotions that foster slow-strategy orientations and buffer the translation of sensation seeking into risky behavior.

Cognitive Dissonance Theory holds that conflict between beliefs and actions induces discomfort, motivating behavior change to restore consonance (Thibodeau and Aronson, 1992). When high sensation seekers who strongly identify with the law engage in risk taking, they experience dissonance that drives reduction of such behavior.

Behavioral Reinforcement Theory posits that compliance with norms yields social approval as secondary reinforcement (Chen and Liu, 2007). Positive legal emotions amplify the salience of this reinforcement, counteracting the allure of risk taking and steering even high sensation seekers toward safer choices.

This study found that negative legal emotions did not moderate the relationship between sensation seeking and risk-taking behaviors among college students, contrary to our hypothesis. While event-related emotions—such as heartbreak—can acutely influence risk choices (Hong et al., 2015), emotional responses are generally divided into inherent moods, shaped by past experiences, and activated emotions, triggered by immediate contexts (Ellen et al., 2006). Our self-report questionnaire captured mainly the former—stable negative legal moods formed through prior encounters with the legal system. Negative legal emotions typically involve cognitive appraisals of institutional trust, fairness, and justice violations, which engage in-depth reflective processing and may not readily interact with sensation-seeking drives to alter risk decisions. In contrast, activated negative legal emotions—elicited by specific legal events—are more likely to affect momentary decision-making. Additionally, enduring mood states in everyday contexts often have minimal impact on novel task processing (Chen and Li, 2006), further explaining the absence of a moderating effect. In contrast, positive legal emotions—being more readily activated by daily affirmations of legal fairness and safety—operate through a security → competence → autonomy chain, enabling even high sensation seekers to exert greater impulse control and choose non-risky actions.

### Limitations and future directions

First, the internal consistency of the Brief Sensation Seeking Scale was relatively low (Cronbach’s *α* = 0.67), which may have introduced measurement error and weakened the strength of observed associations. Future research should consider using more reliable scales to enhance measurement accuracy. Second, negative legal emotions were assessed through self-report measures, which primarily reflect stable, trait-like emotional tendencies rather than situational emotional states. This may limit their ability to interact with motivational variables like sensation seeking. Future studies could use experimental methods to induce state-based legal emotions and better assess their moderating effects.

## Data Availability

The original contributions presented in the study are included in the article/supplementary material, further inquiries can be directed to the corresponding authors.

## References

[ref1] Aslan A. Dinc D. Kutuk B. (2017). “Joint effects of anxiety and mood induction on risk-taking behavior for elderly and young” In eds. M. Gammone, M.A. Icbay, and H. Arslan Recent developments in education, E-BWN. 471–478.

[ref2] Ben-Zur H. Zeidner M. (2009). Threat to life and risk-taking behaviors: a review of empirical findings and explanatory models. Personal. Soc. Psychol. Rev. 13, 109–128. doi: 10.1177/1088868308330104, PMID: 19193927

[ref3] Blair R. J. R. (2017). Emotion-based learning systems and the development of morality. Cognition 167, 38–45. doi: 10.1016/j.cognition.2017.03.013, PMID: 28395907 PMC5572654

[ref4] Chein J. Albert D. O'Brien L. Uckert K. Steinberg L. (2011). Peers increase adolescent risk-taking by enhancing activity in the brain’s reward circuitry. Dev. Sci. 14, F1–F10. doi: 10.1111/j.1467-7687.2010.01035.x 21499511 PMC3075496

[ref5] Chen L. Li W. (2006). The influence of mood state on processing of emotional information. Psychol. Explor. 26, 36–41. doi: 10.3969/j.issn.1003-5184.2006.04.008

[ref6] Chen X. Li F. Nydegger L. Gong J. Ren Y. Dinaj-Koci V.. (2013). Brief sensation seeking scale for Chinese: cultural adaptation and psychometric assessment. Pers. Individ. Differ. 54, 604–609. doi: 10.1016/j.paid.2012.11.007, PMID: 23316097 PMC3539791

[ref7] Chen Q. Liu R. (2007). Contemporary psychology of education. Beijing: Beijing Normal University Press, 83–97.

[ref8] Cheng A. S. K. Ng T. C. K. Lee H. C. (2012). Impulsive personality and risk-taking behavior in motorcycle traffic offenders: a matched controlled study. Pers. Individ. Differ. 53, 597–602. doi: 10.1016/j.paid.2012.05.007

[ref9] Cui L. Colasante T. Malti T. Ribeaud D. Eisner M. P. (2016). Dual trajectories of reactive and proactive aggression from mid-childhood to early adolescence: relations to sensation seeking, risk taking, and moral reasoning. J. Abnorm. Child Psychol. 44, 663–675. doi: 10.1007/s10802-015-0079-7, PMID: 26370547

[ref10] Deci E. L. (1971). Effects of externally mediated rewards on intrinsic motivation. J. Pers. Soc. Psychol. 18, 105–115. doi: 10.1037/h0030644

[ref11] Devlin H. C. Johnson S. L. Gruber J. (2015). Feeling good and taking a chance? Associations of hypomania risk with cognitive and behavioral risk taking. Cogn. Ther. Res. 39, 473–479. doi: 10.1007/s10608-015-9679-3

[ref12] Dippong J. Fitch C. (2017). Emotions in criminological theory: insights from social psychology. Sociol. Compass 11:e12473. doi: 10.1111/soc4.12473, PMID: 40612922

[ref13] Do K. T. Sharp P. B. Telzer E. H. (2020). Modernizing conceptions of valuation and cognitive control deployment in adolescent risk-taking. Curr. Dir. Psychol. Sci. 29, 102–109. doi: 10.1177/0963721419887361, PMID: 33758473 PMC7984409

[ref14] Duell N. Steinberg L. Chein J. Al-Hassan S. M. Bacchini D. Lei C.. (2016). Interaction of reward seeking and self-regulation in the prediction of risk taking: a cross-national test of the dual systems model. Dev. Psychol. 52, 1593–1605. doi: 10.1037/dev0000152, PMID: 27598251

[ref15] Ellen P. Daniel V. Tommy G. Paul S. (2006). Affect and decision making: a “hot” topic. J. Behav. Decis. Mak. 19, 79–85. doi: 10.1002/bdm.528

[ref16] Ferrer R. A. Maclay A. Litvak P. M. Lerner J. S. (2017). Revisiting the effects of anger on risk-taking: empirical and meta-analytic evidence for differences between males and females. J. Behav. Decis. Making 30, 516–526. doi: 10.1002/bdm.1971

[ref17] Hong S. Xiumin D. Zhigang Y. Yaowu S. Psychology D. O.. (2015). The influence of breaking-up mood and breaking-up emotion priming on risk-taking behavior. J. Psychol. Sci. 38:6. doi: 10.16719/j.cnki.1671-6981.2015.02.014

[ref001] Jessor R. Jessor S L. (1977). Problem behavior and psychosocial development: A longitudinal study of youth[J]. New York: Academic Press. 7, 948–949. doi: 10.2307/2065689

[ref19] Jianhua H. Su X. Shuhui X. (2025). Parent–child attachment and adolescent problematic behavior: the mediating effect of legal emotions. Front. Psychol. 16:1546895. doi: 10.3389/fpsyg.2025.1546895, PMID: 40083762 PMC11903415

[ref20] Lejuez C. W. Read J. P. Kahler C. W. Richards J. B. Brown R. A. (2002). Evaluation of a behavioral measure of risk-taking: the Balloon Analogue Risk Task (BART). J. Exp. Psychol. Appl. 8, 75–84. doi: 10.1037/1076-898X.8.2.75, PMID: 12075692

[ref21] Li A. Tan L. Sun H. Xiong G. Pan J. (2016). The effect of sleep deprivation on risky choice: a dual-process models approach. Adv. Psychol. Sci. 24, 804–814. doi: 10.3724/SP.J.1042.2016.00804

[ref22] Liang S. Yang G. (2024). Poverty leads to the desire to change, and wealth leads to the desire for stability: the impact of perceived money scarcity and abundance on individual risk decision-making. Adv. Psychol. Sci. 32, 1233–1249. doi: 10.3724/SP.J.1042.2024.01233

[ref23] Liu S. Fan H. Wang Z. Song M. Teng H. (2025). Parental marital conflict and bullying among middle school students: roles of deviant peer affiliation and sensation seeking. Psychol. Dev. Educ. 41, 235–244. doi: 10.16187/j.cnki.issn1001-4918.2025.02.09

[ref24] Lydon-Staley D. M. Falk E. B. Bassett D. S. (2020). Within-person variability in sensation-seeking during daily life: positive associations with alcohol use and self-defined risky behaviors. Psychol. Addict. Behav. 34, 257–268. doi: 10.1037/adb0000535, PMID: 31815502 PMC7064376

[ref25] Lydon-Staley D. M. Geier C. F. (2017). Age-varying associations between cigarette smoking, sensation seeking, and impulse control through adolescence and young adulthood. J. Res. Adolesc. 28, 354–367. doi: 10.1111/jora.12335, PMID: 28891119 PMC5845819

[ref26] Mann F. D. Engelhardt L. Briley D. A. Grotzinger A. D. Harden K. P. (2017). Sensation seeking and impulsive traits as personality endophenotypes for antisocial behavior: evidence from two independent samples. Pers. Individ. Differ. 105, 30–39. doi: 10.1016/j.paid.2016.09.018, PMID: 28824215 PMC5560504

[ref27] Ming L. I. Man-Wai L. I. Wen-Qiao L. I. Ding-Guo G. (2019). The effect of awe on ethical risk-taking propensity. Chin. J. Appl. Psychol. 25, 48–58. doi: 10.3785/1006-6020.2019.01.0048

[ref28] Rui D. Kaiping P. Feng Y. (2013). Positive emotion: awe. Adv. Psychol. Sci. 21, 1996–2005. doi: 10.3724/SP.J.1042.2013.01996

[ref29] Shenhav A. Botvinick M. Cohen J. (2013). The expected value of control: an integrative theory of anterior cingulate cortex function. Neuron 79, 217–240. doi: 10.1016/j.neuron.2013.07.007, PMID: 23889930 PMC3767969

[ref30] Shi L. Kan S. Hui Z. Bin W. Yue H. U. (2019). Effect of parental behavioral control on water high-risk practices for adolescents: moderated mediating effect. Stud. Psychol. Behav. 17, 259–267. doi: 10.3969/j.issn.1672-0628.2019.02.016

[ref31] Shulman E. P. Harden K. P. Chein J. M. Steinberg L. (2016). The development of impulse control and sensation-seeking in adolescence: independent or interdependent processes? J. Res. Adolesc. 26, 37–44. doi: 10.1111/jora.12181

[ref32] Simpson J. A. Griskevicius V. Kuo S. I. Sung S. Collins W. A. (2012). Evolution, stress, and sensitive periods: the influence of unpredictability in early versus late childhood on sex and risky behavior. Dev. Psychol. 48, 674–686. doi: 10.1037/a0027293, PMID: 22329381

[ref33] Steinberg L. (2017). A social neuroscience perspective on adolescent risk-taking. Dev. Rev. 28, 78–106. doi: 10.1016/j.dr.2007.08.002 PMC2396566 18509515

[ref34] Tang S. Zheng Y. He J. (2022). Automatic processing advantages of high sensation seekers for risk sounds: evidence from N1, P2, and MMN. J. Psychol. Sci. 45, 1344–1351. doi: 10.16719/j.cnki.1671-6981.20220609

[ref35] Thibodeau R. Aronson E. (1992). Taking a closer look: reasserting the role of the self-concept in dissonance theory. Personal. Soc. Psychol. Bull. 18, 591–602. doi: 10.1177/0146167292185010

[ref36] Xu S. Yan W. (2022). The development characteristics, influencing factors and mechanism of adolescents’ legal consciousness. Zhejiang: Zhejiang University Press, 110–116.

[ref37] Xu S. Yu J. Fan L. Yang Q. Wang Z. Zhang Y. (2024). The influencing factors of college students' legal emotion and the mechanism of its effect on aggressive behavior. Front. Psychol. 15:1295915. doi: 10.3389/fpsyg.2024.1295915, PMID: 38699570 PMC11063308

[ref38] Yan C. (2012). The history of western legal thoughts. Beijing: Law Press China, 242–243.

[ref39] Yu T. Hu J. (2023). A review of adaptive and maladaptive risk-taking behavior among adolescents. J. Psychol. Sci. 46, 1440–1446. doi: 10.16719/j.cnki.1671-6981.20230621

[ref40] Zuckerman M. (1983). Sensation seeking and sports. Pers. Individ. Differ. 4, 285–292. doi: 10.1016/0191-8869(83)90150-2

